# PZT/PZT and PZT/BiT Composite Piezo-Sensors in Aerospace SHM Applications: Photochemical Metal Organic + Infiltration Deposition and Characterization

**DOI:** 10.3390/s19010013

**Published:** 2018-12-20

**Authors:** Hamidreza Hoshyarmanesh, Nafiseh Ebrahimi, Amir Jafari, Parisa Hoshyarmanesh, Minjae Kim, Hyung-Ho Park

**Affiliations:** 1 Project neuroArm, Department of Clinical Neurosciences and Hotchkiss Brain Institute, Cumming School of Medicine, University of Calgary, Calgary, AB T2N 4Z6, Canada; hamidreza.hoshyarman@ucalgary.ca; 2 Department of Mechanical Engineering, University of Texas at San Antonio, One UTSA Circle San Antonio, San Antonio, TX 78249, USA; nafiseh.ebrahimi@utsa.edu (N.E.); amir.jafari@utsa.edu (A.J.); 3 Department of Organic Chemistry, University of Isfahan, Isfahan 81746-73441, Iran; p.hoshyarmanesh@gmail.com; 4 Department of Materials Science and Engineering, Yonsei University, Seoul 03722, Korea; minjae_kim@yonsei.ac.kr

**Keywords:** composite, piezoelectric sensor, thick film, sol-gel PMOD deposition, infiltration, PZT/PZT, PZT/BiT, characterization, structural health monitoring, aerospace structure

## Abstract

The composition of fine-ground lead zirconate-titanate powder Pb(Zr_0.52_Ti_0.48_)O_3_, suspended in PZT and bismuth titanate (BiT) solutions, is deposited on the curved surface of IN718 and IN738 nickel-based supper alloy substrates up to 100 µm thickness. Photochemical metal organic and infiltration techniques are implemented to produce smooth, semi-dense, and crack-free random orientated thick piezoelectric films as piezo-sensors, free of any dopants or thickening polymers. Every single layer of the deposited films is heated at 200 °C with 10 wt.% excess PbO, irradiated by ultraviolet lamp (365 nm, 6 watt) for 10 min, pyrolyzed at 400 °C, and subsequently annealed at 700 °C for one hour. This process is repeated successively until reaching the desired thickness. Au and Pt thin films are deposited as the bottom and top electrodes using evaporation and sputtering methods, respectively. PZT/PZT and PZT/BiT composite films are then characterized and compared to similar PZT and BiT thick films deposited on the similar substrates. The effect of the composition and deposition process is also investigated on the crystalline phase development and microstructure morphology as well as the dielectric, ferroelectric, and piezoelectric properties of piezo-films. The maximum remnant polarization of *P_r_* = 22.37 ± 0.01, 30.01 ± 0.01 µC/cm^2^, the permittivity of *ε_r_* = 298 ± 3, 566 ± 5, and piezoelectric charge coefficient of *d_33_* = 126, 148 m/V were measured versus the minimum coercive field of *E_c_* = 50, 20 kV/cm for the PZT/PZT and PZT/BiT thick films, respectively. The thick film piezo-sensors are developed to be potentially used at frequency bandwidth of 1–5 MHz for rotary structural health monitoring and also in other industrial or medical applications as a transceiver.

## 1. Introduction

Piezoelectric thin films have been used vastly as sensors, accelerometers, micro-motors, and many other low-force electromechanical (E/M) applications [1]. However, high-frequency transducers for structural health monitoring and vibration control systems usually require higher thicknesses to convey E/M stimulation loads appropriately [2,3,4,5,6,7,8,9]. Therefore, thin films cannot be applied this way. Ferroelectric thick films for applications, such as high-frequency sonars [10] micro-electromechanical devices [11], elastic surface wave generators [12], hydrophones [13], and sensors [14], have been of great interest in the last decade; since such films have both characteristics of the bulk material and thin-layer films at the same time [15]. In this regard, the process of thick films is a developing area of research. Nevertheless, there still exists a significant lack of integrated thick piezoelectric films specifically developed for continuous structural health monitoring of rotary structures in the aerospace industry. Conventional methods currently being used for the production of thick films, including pulsed laser deposition [16,17], print screen [18], sol-gel spray [19], and hybrid sol-gel [14,20,21], are not effective nor economical to produce dense films in a rather short time with minimal waste material, particularly for deposition on substrates with irregular shapes. Deposition of thick films using sol-gel method entails a significant number of repetitions to get the desired thickness, layer-after-layer. This leads to frequent temperature fluctuations that might increase the possibility of cracking due to film contraction or thermal fatigue stress at each layer [22]. Among the aforementioned methods, the hybrid sol-gel has been taken more into consideration for thick films due to low preparation time, cost-effectiveness, and appropriate controllability on the precursor’s stoichiometry [20]. The aforementioned may not be so easy to achieve through other methods due to prolonged processing time (slow sedimentation rate), lack of control of compound’s stoichiometry, and conductivity issues [23]. Reactive Magnetron Pulsed DC Sputtering (RMPDS) or RF-Bias might be a good solution; nonetheless, it is not yet an appropriate system capable of coating over curved surfaces of large industrial components.

The sol-gel is an appropriate method to prepare thick ceramic films with the thickness of more than one micron. The fabrication of thick composite films with this method has two main steps (1) sol-gel matrix selection, and (2) dispersion of the piezoelectric powder within the sol-gel matrix. During the second step, preparation of a homogenous suspended mixture is important to reduce the possibility of the agglomeration [21,22,23,24,25]. Attention should be given to inappropriate mixing of the powder within the piezoelectric solution and length of time before mixture deposition, which could possibly yield to (1) forming the surface layers, (2) strengthening the surface tension forces, (3) sedimentation of the suspended powder, and, therefore, (4) agglomeration after the coating process. To avoid the agglomeration before and during the preparation process of thick films, researchers usually apply high molecular-weight solvents, such as acetic acid (60.05 g/mol), hexane (86.17 g/mol), methoxyethanol (76.09 g/mol), and α-terpined (154.25 g/mol) [1], binders including polyvinylpyrrolidone, plasticizers such as polyethylene glycol and organic diffusers, such as buthoxyethoxy-ethyl acetate. Although the smaller particles seem to result in less localized powder accumulation in the sol-gel matrix and improve the density of the resultant film, this perspective is not entirely true. The reduction of the powder size leads to an increase in the effective particle surface, which raises the surface tension. The surface tension, remarkably, contributes in the powder agglomeration. Therefore, the conventional solid-state oxide method (dry method) with particle sizes larger than 2 μm for the production of PZT powder is preferred vs. a coprecipitation process (wet method), which normally causes particle sizes smaller than 2 μm in diameter. An ultrasound vibrator was employed to disperse the piezoelectric powder in the precursor solution to avoid agglomeration and consequently reduce the porosity of thick films [20].

Barrow et al. [25] fabricated thick piezoelectric films without cracks. They attributed the crack-free feature of the proposed films to the following factors: (1) a considerable powder to precursor solution ratio, which reduces the shrinkage after annealing, and (2) a strong bound between sol-gel and PZT particles. Wu et al. [26] declared that the presence of 1% wt PZT powder in the sol-gel has more advantages, e.g., a 50-degree reduction in the temperature of the perovskite phase formation and a significant improvement in dielectric and ferroelectric characteristics. Nevertheless, many researchers believe that the intermixture of piezoelectric powder with sol-gel matrix causes non-homogeneous crystallization of the perovskite phase and randomly oriented structures.

In this research, PZT/PZT and PZT/bismuth titanate (BiT) composite thick films are deposited on the curved surface of IN718 and IN738 superalloy substrates using photochemical metal organic deposition including ultraviolet (UV) irradiation and infiltration. The details of the deposition process will be presented in Section 2. The experimental set up will be introduced in Section 3. Section 4 involves the subsequent results of characterization regarding the microstructural, morphological, dielectric, ferroelectric, and piezoelectric properties of the proposed thick films. Section 5 is assigned to the conclusions.

## 2. Materials and Methods

A hybrid sol-gel technique, PMOD and Infiltration, was applied in this research to deposit piezo-transducers in the form of composite thick films on the curved surface of industrial components. Taking advantage of this hybrid method, PZT/PZT and PZT/BiT composites were prepared with the introduction of a certain mass percentage of PZT-5A powder, with 2–5 μm particle size, in PZT and BiT precursor solutions, respectively. Piezoelectric characteristics of PZT-5A is presented in Table 1 [27,28,29]. The dielectric constant *ε_r_* in the tables is correlated with free piezoelectric samples. The stacked wafers bonded to a substrate mostly have *ε_r_* < 1000 [30].

PZT precursor solution was prepared using lead 2-ethylhexanoate, zirconium 2-ethyl hexanoate, and Ti-isopropoxide in molar ratio Pb:Zr:Ti = 1.1:0.52:0.48 as the precursors. The applied mixing order and synthesis process were the same as proposed in Reference [4,6,7]. Then, hexane solvent was added to the solution to dilute it to 0.4 M. To make the BiT precursor solution of molar ratio Bi:Ti = 4.4:3, bismuth 2-ethylhexanoate was dissolved in hexane as the solvent. Then titanium isopropoxide was added, and the mixture was stirred on the vibrator for 4 h, as described in Reference [4,6,7]. Before starting off the coating process, the substrates were washed and shadow masked in square shapes of 10 × 10 mm and 15 × 15 mm using temperature-resistant Kapton tape. Two layers of the 600-nm gold bottom electrode were deposited on the masked surface using a thermal evaporation system. Coating of composite films was initiated by the injection of the suspended mixture of PZT/PZT and PZT/BiT onto the surface of Si and Pt/Si flat wafers.

**Figure 1:**
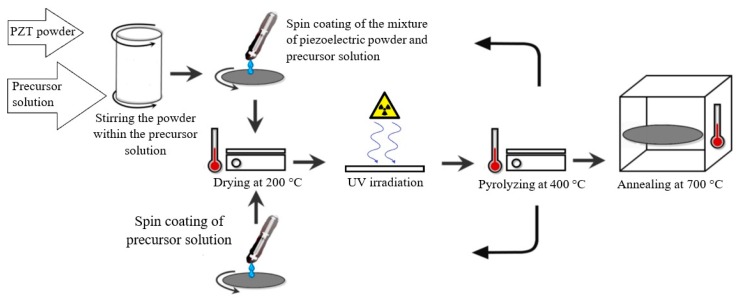
The consecutive steps of hybrid sol-gel deposition including the mixture of piezoelectric powder and precursor solution, injection, drying, ultra violet (UV) irradiation, pyrolyzing, and annealing.

Each deposited layer then went through drying at 200 °C on a hot plate for 2 min, UV irradiation inside an exposure chamber under a 365 nm UV lamp for 10 min, and pyrolyzation at 400 °C for 2 min, successively. These procedures were repeated for the subsequent layers to reach the desired thickness. The rotational speed of the spin coater was adjusted at 1500 rpm, much less than that is usually used for thin and thick films. Higher speeds cause the piezoelectric powder to sprinkle out entirely and do not make a uniform layer. The UV irradiation allowed for a better rupture and ejection of the hydrocarbon chains of the ligands from the precursor films. A scheme for the consecutive steps of the hybrid sol-gel process is illustrated in Figure 1.

**Figure 2:**
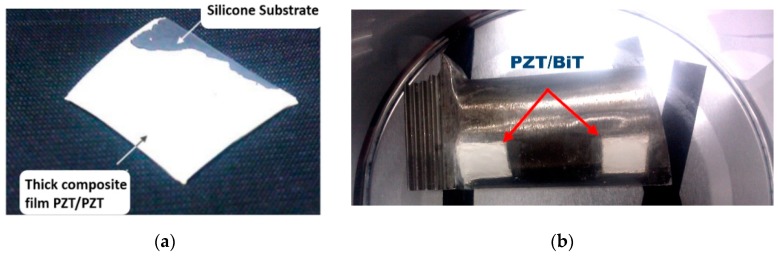
(**a**) 5-layer PZT/PZT on flat silicon substrate and (**b**) 5-layer PZT/bismuth titanate (BiT) composite film on curved superalloy substrate, both derived from PMOD sol-gel method.

Figure 2 shows two samples of composite PZT/PZT and PZT/BiT thick films deposited on the surface of a flat silicon wafer and a curved superalloy turbine blade using photochemical sol-gel technique. The results reveal that sol-gel deposition of the thick composite films lacks enough bonding to the substrate, durability, strength, smoothness, and resistance to cracking. To avoid the aforementioned shortcoming regarding the deposition of thick piezoelectric films using the sol-gel technique, the infiltration method was implemented. To apply the infiltration technique, the injection was modified such that the PZT or BiT precursor solution was initially dropped down as the first layer followed by the injection of the mixture of PZT/PZT or PZT/BiT suspension upon the surface of Si and Pt/Si flat wafers. These two steps, i.e., deposition of the precursor solution as the odd layers and powder suspension deposition as the even layers, were cyclically repeated for the subsequent layers.

The surface morphology and microstructure of the resultant films were recorded by a scanning electron microscope (SEM) model FESEM JEOL JSM-600F (JEOL, Pleasanton, CA, USA). The surface profile and the thickness of the films were measured by Alpha-Step IQ (KLA-Tencor, Milpitas, CA, USA). The surface roughness of the samples was investigated using Perthometer M2 (Mahr, Esslingen, Germany). The grain size, phase, and crystalline microstructure of the films were also measured and recorded using Rigaku D/MAX-2000 X-ray diffractometer (Rigaku, Tokyo, Japan) with the diffraction range of 20–80° at 40 kV, 30 A, 4 deg./min and 0.02° angular resolution.

## 3. Design and Experiments

To coat the thick layers of piezoelectric films on the curved surface of superalloy blades IN718 and IN738 as the main substrates in this research, the blades were first washed and masked similar to the previous explanation. We washed the blades using acetone and methanol and put them in the ultrasonic bath of ethanol and deionized water for a longer time compared to the small Si and Pt wafers. Then, a layer of PZT or BiT precursor solution was deposited on the top surface of the blades using a 0.3-micron filter. The injection was carried out over the same area already coated with a gold bottom electrode. After injection of each layer, the blades were placed in the spin coater to disperse the localized drop uniformly over the whole masked area at 1500 rpm for 30 s. The procedures of UV irradiation, pyrolyzing, and annealing was thoroughly performed for the first layer to the end.

After the suspended mixture of PZT/PZT or PZT/BiT composite was dropped down over the first layer and the composite layer was taken into the post-injection procedures, similar to the first one. To reach the composite structure of *n* [C + *k*S] in this fashion, the films were made *n* mixed layers, each of which comprises one composite PZT/PZT or PZT/BiT (C) layer in addition to *k* layers of the PZT or BiT precursor solutions (S). In other words, along with each layer of PZT/PZT or PZT/BiT, *k* layers of a sol-gel solution was injected. This approach was taken to first, eliminate the graded structure which causes the reduction of dielectric, ferroelectric, and piezoelectric characteristics, and second, to homogenize the layer structure and effect a diminution in the porosity of the thick composite films. In this research, *k* = 2 opted.

The rotational speed of the spin coater was proportionally adjusted to the average PZT particle size and the viscosity of the sol-gel matrix. With larger the average particles size and more viscose the solution, the rotational speed decreased toward 1000 to 1500 rpm. By reducing the grain size, the appropriate speeds in the range of 1500 to 4000 could be selected proportional to the final thickness. The 20–30 s for the spinning time worked appropriately because below 20 s the piezoelectric powder is not going to be scattered entirely and evenly on the substrate, and the timing beyond the 30 s does not improve the outcome. The resultant film after pyrolysis had a proper macroscopic quality and had no visual cracks. It also had an even thickness accompanied by an almost homogeneous structure, white in color.

The first layer, which was the interface between the piezo-film and the substrate, and the last layer, the top surface of the film, was coated with PZT or BiT sol-gel solution. The aim of the former is to strengthen the connection between the composite piezo-ceramic film and the substrate; the latter is performed to reduce the surface roughness and minimize the wear percentage as much as possible. As the number of layers increased, the previous composite layers were enriched with a sol-gel solution, which filled the cavities of the underlying layers and reduced the porosity. Cruz [32] had already recognized the saturation capacity of this repetition as up to four times for sol-gel penetration. According to him, iteration does not affect the porosity of the underlying layers after four of infiltration procedures are conducted. Ball milling the PZT powder for 60 h and using the ultrasonic vibrator for extracting grain size of 2 microns helped avoid the additional adhesives, plasticizers, and solvents. Hence, in addition to reducing the impurities, the length of time spent on drying and pyrolyzing of thick piezo-ceramic films—that sometimes exceeding 24 h—was considerably reduced [1]. Refraining from using solvents with a high molecular mass leads to a reduction of the formation of large porosities during the evaporation of volatile substances.

Thermal treatment and post-processing of thick films after coating in the hybrid sol-gel method does not differ much from the method implemented for thin films, mentioned in Reference [33]. One major difference is the cooling rate of the samples after drying, pyrolyzing, and annealing. The cooling rate of 5 °C/min showed promising results in eliminating surface cracking. Other researchers have implemented a drying temperature region of 100–300 °C for one to several minutes, depending on the type of solvent and also the organic compounds in the sol-gel matrix. Moreover, the pyrolyzing process—also known as pre-annealing or calcination—was carried out in the temperature range of 300–500 °C for one to several minutes. Due to the curvature of the superalloy blades and the lack of direct contact with the hot plate, uniform distribution of thermal energy over the whole surface of the blade was not feasible. Thus, during the spinning, drying, and pyrolyzing processes, aluminum foil pads with the same curvature as the blades were located underneath with the aim of effecting a uniform distribution of thermal energy all over the piezoelectric films. The annealing process was accomplished for the formation of the perovskite crystal phase in piezo-ceramic samples. The annealing temperature for the PZT/PZT and PZT/BiT films was adjusted at 700 °C and the annealing time was confined to 60 min similar to what was reported for thin films [23,32,34]. Considering that spin coating is a very common method for the flat and small substrates, spin coating of composite piezo-ceramic films on a curved substrate is challenging. The smaller Ruston-TA blade, made of IN738, with 60 mm length, 30 mm width, and 52 g mass was coated first. The greater challenge was for coating the JT8D blades, made of IN718, with 128 mm length, 46 mm width, and 212 g mass. The spin coating on this blade looked practically unfeasible. For this reason, we considered an injection method for this type of substrate and the spinning step was eliminated.

**Figure 3:**
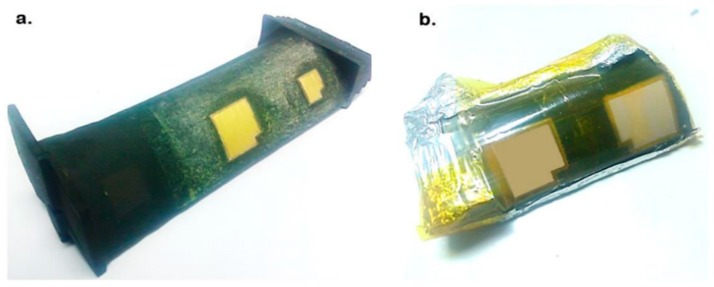
Deposition of the composite piezoelectric films on the curved surface of superalloy blades using the hybrid sol–gel-infiltration method, (**a**) JT8D L_1_ and (**b**) Ruston-TA S_5_.

Six JT8D, hereafter called L_1_–L_6_, and five Ruston-TA, S_1_–S_5_, blades were considered for the deposition process. Figure 3a depicts a couple of 10 × 10 mm and 15 × 15 mm semi-square PZT/BiT deposited films with 100 μm thickness on the blade L_1_ at positions A (near the root) and B (center), respectively. Figure 3b shows two 15 × 15 mm PZT/PZT films of 30 μm thickness at positions A and C (near the shroud) on the blade S_5_ applying the hybrid sol-gel deposition method. A small part of each film remained uncoated to provide access to the bottom gold electrode for micro-wiring and post electrical characterization of the piezo-sensors. In the end, two layers of the 600-nm thick gold electrode were coated on top of each film, deposited on the blades of type L, using the evaporation method. Five layers of platinum with the final thickness of 100 nm were also deposited over the composite films, laid on the blades of type S, using a DC sputtering method.

After coating the top electrode, the samples underwent a secondary annealing in the tube furnace for 30 min at 600 °C to make a proper connection between the electrodes and the piezoelectric films. The thickness of the top electrode had an optimal value in terms of maximum electrical conductivity and minimum impact on the electromechanical properties of the piezoelectric films. It is worth noting that the ohmic behavior of the films decreases as their planar dimensions increase [35]. In thicker electrodes, the resistance is less affected by the electrode width. However, the growth of electrode thickness has a negative impact on the mechanical behavior of the composite piezo-sensors and piezo-actuators. The 600 nm seems an appropriate value regarding the surfaces roughness of the films and the height of asperities.

## 4. Results and Discussions

### 4.1. Visual Microscopic Inspection

**Figure 4:**
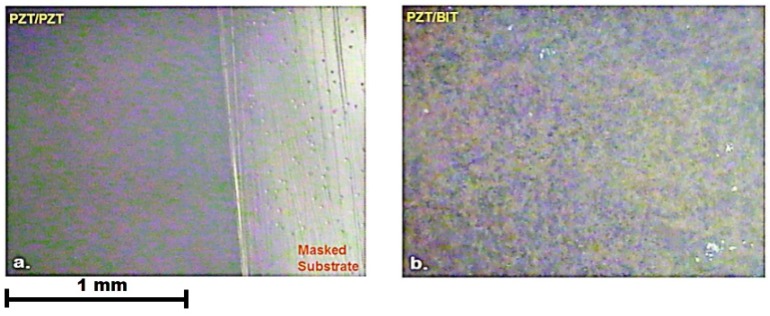
Optical microscopic images, composite films of (**a**) PZT/PZT on S_5_ and (**b**) PZT/BiT on L_5_.

The effect of intermittent injection of sol-gel solution among the composite layers on the structure of the thick PZT/PZT and PZT/BiT films is first studied by analyzing the optical microscopic images presented in Figure 4. The figure depicts that the penetration of sol-gel solution was very effective in the fabrication of a crack-free and smooth surface, with reduced porosity and a strengthened cohesion of the films compared with the samples for which infiltration had not been used. The images were captured from the film boundaries, where the agglomeration, cracks, voids, pores, and thickness non-uniformity are more likely, compared to the central areas.

### 4.2. Surface Morphology

The mean value for the micro-hardness of the samples, out of three iterations for each sample, was measured via Vickers Indentation method and shown in Table 2. Since such films are supposed to be utilized actively in real-time structural health monitoring of rotary structures, the rotational speed of the blades causes abrasive wear and erosion especially in the field applications that might result in deterioration of top electrodes and piezoelectric films. Using the infiltration method it is not only possible to control of surface roughness of the composite films but also to enhance the density and surface hardness of piezoelectric films to a great extent by filling the porosity of the surface to a high percentage. As inferred from Table 2, the film density affects the micro-hardness of piezoelectric films, significantly. By a reduction in porosity, micro-hardness will show a sensible rise. PZT is structurally harder than BiT; previous authors reported the Vickers hardness of reinforced BiT with zirconium and sodium elements as 3.2–5.4 GPa [36]. It is expected that the additive-free composite films consisting of BiT ingredients would have rather less hardness. In addition, the hardness of additive-free samples of PZT has been reported as 3.4 GPa with 95% density under a load of 500 g [37,38].

The average roughness (*R_a_*) of composite thick films was also measured as represented in Table 3. *R_a_* is a function of the grain size, the film thickness, and the number of sol-gel infiltration steps. It grows proportionally with the increase in the number of layers and also the film thickness. The thick films mostly have a higher *R_a_* due to encompassing larger particles compared to thin films. The smaller the *R_a_*, the narrower and sharper the peaks in the X-ray diffraction patterns as addressed in Reference [1]. The sol-gel infiltration shows a great contribution to the improvement of the surface roughness, electrode connection, and the precision of the profilometry.

**Figure 5:**
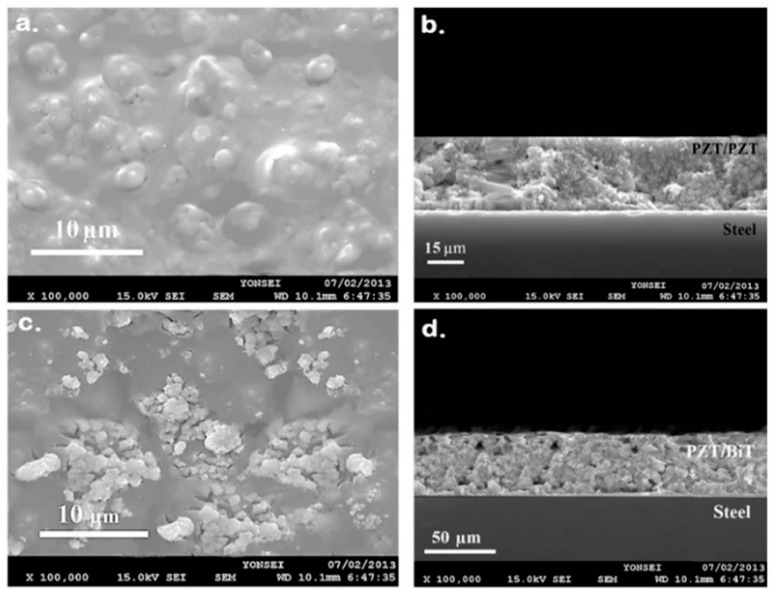
SEM images captured from hybrid thick films of type 5[C + 2S] (**a**,**b**) PZT/PZT of 30 μm thickness, and (**c**,**d**) PZT/BiT of 50 μm thickness.

Figure 5 shows the SEM images captured from the thick composite films of PZT/PZT and PZT/BiT of type 5[C + 2S] with a thickness of 30 and 50 microns, respectively. The images illustrate the uniform distribution of PZT powder in the sol-gel matrix and a smooth surface on the top layer. Increasing the concentration of the precursor solution to above 0.4 M boosts the risk of trapped bubble formation in the piezoelectric film. Thereupon, the final concentration of the mixture was considered as 0.4 M. Similar results regarding the concentration of PZT composite films were already reported by Dorey et al. [39] who studied the effects of spin coating and subsequently repeated infiltration of PZT solution using Cu_2_O-PbO sintering aid. However, in the present research, the coating process of composite PZT/PZT and PZT/BiT thick films on the curved surface of superalloy blades was carried out free of spin coating, additives, and sintering aid.

### 4.3. Crystalline Structure

**Figure 6:**
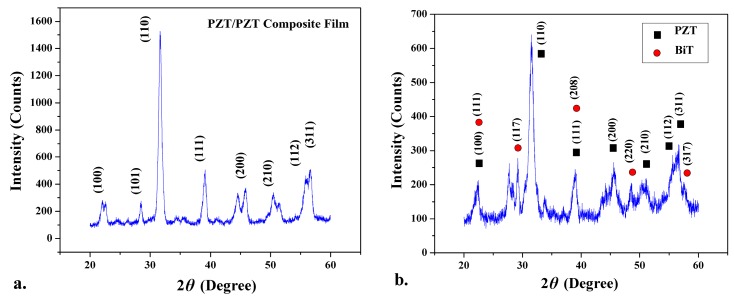
X-ray diffraction (XRD) patterns for (**a**) PZT/PZT and (**b**) PZT/BiT thick composite films.

The X-ray diffraction (XRD) patterns obtained from multilayer PZT/PZT and PZT/BiT composite films are presented in Figure 6. The relatively higher intensity of PZT (110) is related to piezoelectric powder PZT-5A incorporated in the texture of the PZT/PZT and PZT/BiT films. The orientations of (100), (101), (111), (200), (210), (112), and (311) obtained for PZT in Figure 6a were also observed for the thick PZT films not mixed with powder in Reference [7]. In Figure 6b, black squares correspond to the PZT powder within the texture of sol-gel derived BiT crystals, which show the same planes as obtained for the sol-gel derived PZT crystals in Figure 6a. The planes (111), (117), (208), (220), and (317) indicated by red circles represent the sol-gel derived BiT crystals. The graph shows no impact of BiT on crystallization of PZT after annealing. XRD graphs indicate the formation of no secondary phases during multiple repetitive annealing procedures, which reflect the quality of the resultant crystalline structure in terms of purity, sensitivity, and stoichiometric combinations.

### 4.4. C-V Hysteresis Loop

**Figure 7:**
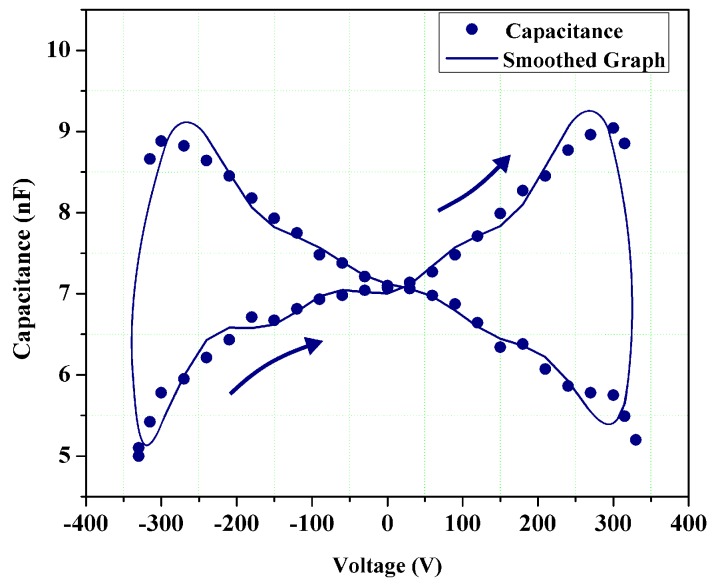
Capacitance-voltage (*C-V*) hysteresis loop of L_1_-B thick film sample according to the Sawyer-Tower method.

The capacitance-voltage (*C-V*) hysteresis loop of several composite samples was calculated using the famous Sawyer-Tower method and applying ±330 VDC at 100 kHz at the ambient temperature. Figure 7 demonstrates the results obtained from the 15 × 15 mm PZT/BiT, L_1_-B sample (at the center of the blade L_1_). The diagram shows a butterfly pattern with two peaks. Typically, dielectric materials do not show any change in the capacitance due to a change in the feed input voltage, as the capacitance is a function of geometrical parameters (thickness and surface area). In piezoelectric materials changing the bias and fluctuating voltages cause a change in electrical impedance, sensor volume and consequently the capacitance.

### 4.5. Curie Temperature (T_c_)

**Figure 8:**
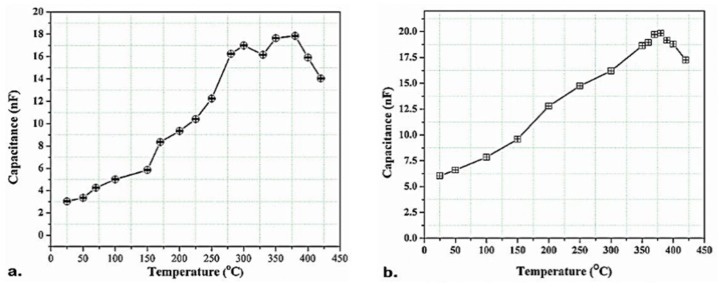
Capacitance-temperature (*C-T*) variations for the PZT/BiT films at 25–420 °C, (**a**) L_1_-A and (**b**) L_1_-B.

**Figure 9:**
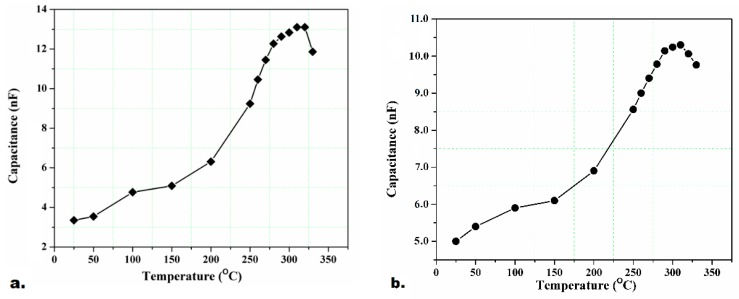
The *C-T* variations for the PZT/PZT films at 25–330 °C, (**a**) L_3_-A and (**b**) L_3_-B.

A number of experiments were carried out to determine the capacitance-temperature (*C-T*) behavior and, therefore, Curie temperature (*T_c_*) of the thick piezoelectric films using a hot plate and the Keithley 590 *C-V* Analyzer in the temperature range of 20–420 °C. Through the tests, the dielectric constant of the samples (*ε_r_*) at various temperatures were obtained, and its maximum value was recorded. At the Curie temperature, the *ε_r_* will be reaching to its maximum. The results expressed the Curie temperature of PZT/PZT samples with 50 μm thickness nearly equal to 300–320 °C, and that of PZT/BiT samples with 100 μm thickness around 370–380 °C. Figure 8 and Figure 9 illustrate the *C-T* diagrams associated with different samples.

### 4.6. Dielectric Permittivity (ε_r_)

**Figure 10:**
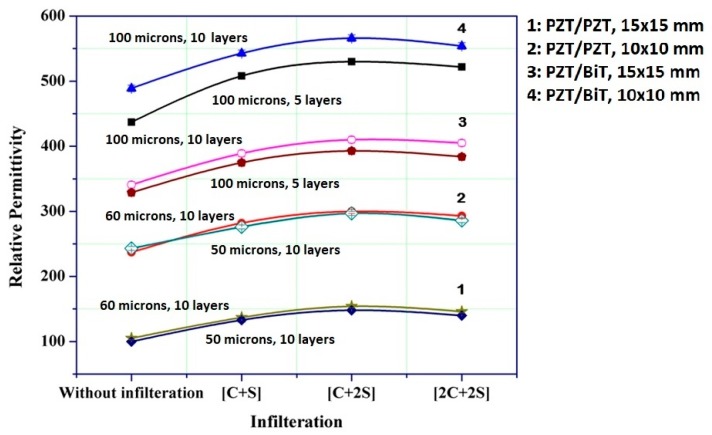
Variation of dielectric permittivity in piezoelectric composite films of [*k*_1_C + *k*_2_S] structure.

According to the acquired results shown in Figure 10, there is a rise in dielectric permittivity (*ε_r_*) as the number of layers, and the infiltration steps increase; nevertheless, the dielectric loss (*tanδ*) remains almost constant. It might happen due to a mitigation in material porosity and improvement in the bonding of piezoelectric powder particles with the sol-gel matrix. Concerning the dielectric permittivity of the samples, porosity acts like series or parallel capacitors in conjunction with the adjacent dense piezoelectric columns. As the volumetric ratio of porous cavities (filled with air) to the piezoelectric film increases, the tendency of the air dielectric permittivity to be placed in serial, versus parallel, with that of the composite film increases. In other words, the volume of porous cavities adversely affects the relative dielectric constant.

The dimension, number of layers, and porosity have a significant impact on relative dielectric constant. For two thick piezo-films with similar dimensions, e.g., L_5_-A and L_5_-B, the dielectric permittivity is almost identical. The dielectric permittivity of PZT/PZT films is less than that of PZT/BiT ones. The difference is due to the better penetration of BiT sol-gel solution among the composite layers which consequently helps of obtaining less porosity. BiT solution also has a higher viscosity compared with PZT solution which makes it easier to reach higher thickness.

The same hold true over the blades of type S such that the S_6_-PZT/BiT films show higher permittivity than S_5_-PZT/PZT films. Except for porosity, another effective parameter on the characteristics of the thick composite films is the volumetric ratio of PZT powder to the sol-gel solution.

### 4.7. Remnant Polarization (P_r_)

**Figure 11:**
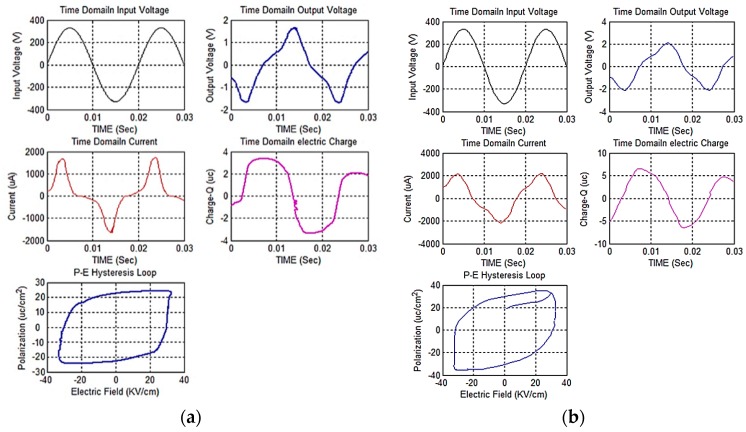
Ferroelectric (*P-E*) hysteresis loop for the composite piezo-films: (**a**) L_1_-A and (**b**) L_1_-B.

The remnant polarization (*P* at electric field = 0), similar to the dielectric permittivity, is raised by increasing the number of sol-gel infiltration through the composite layers. The films of larger dimensions have more desirable conditions in terms of internal tension and uniform heat absorption. Hence, their remnant polarization is a little higher. The results indicate a higher remnant polarization in L-PZT/BiT films compared with the L-PZT/PZT samples. The reason could be attributed to several factors including, better penetration of BiT solution compared to PZT through the composite layers of the thick films, the relative decline in the porosity, higher viscosity of BiT solution that facilitates the deposition of thicker films up to 100 microns in this research. The thicker the films, the greater number of grains will be formed in the microstructure, and the remnant polarization will increase, proportionally. The ferroelectric (*P-E*) hysteresis loops of two samples are displayed in Figure 11. This figure also represents the input (feed) and the amplified output (measured) voltage signals on the oscilloscope during the *P-E* hysteresis loop measurement at 50 Hz. Equation 1 shows how the polarization (*P*) is derived from current (*I*), electric displacement (*D*), electric charge (*Q* = *I*/*t*), time (*t*), cross-section (*A*), and electric field (*E*). The MATLAB coding program for calculation of the *P-E* hysteresis loop is presented in Appendix A, which is based on the standard IEEE180, National Physics Laboratory, UK [40].

(1) $$D=\frac{Q}{A}=\varepsilon E+P$$

### 4.8. Piezoelectric Charge/Strain Coefficients

An APC d_33_ m was employed to measure the longitudinal piezoelectric charge coefficient (*d_33_*) of the thick films. The electrodes laid on the top and bottom (the substrate) of the films were gently clamped to the instrument and d_33_ was measured for some samples. The *d_33_* is a function of the thickness (number of layers), the number of sol-gel infiltrations, and the film porosity. The characterization results of piezoelectric films over the S and L films, represented in Table 4, show that thicker films have greater piezoelectric coefficients due to the larger grain sizes, fewer grain boundaries, less spinning lock on the boundaries, and more freedom of movement on the non-180° domain walls. The larger cross-section and greater number of layers/consecutive thermal treatments lead normally to more porosity and contaminations inside the films and finally a drop in piezoelectric coefficients relative to ideally dense and pure homogenous films. For the cubic stacked piezoelectric samples, the transverse piezoelectric coefficient d_31_ is usually measured by rotating the cubes and clamping the un-electroded faces. The condition of thick piezo-films is much different from stacked piezo-samples. As a result, rotating the piezo-films deposited on turbine blades was a big challenge and the small thickness of the films did not allow the measurement of d_31_.

## 5. Conclusions

PZT/PZT and PZT/BiT composite piezo-sensors were derived in this research from sol–gel-based photochemical metal organic and infiltration technique. Characterization showed promising results in deposition of thick intelligent sensors on the curved surface of heavy superalloy blades, as an example of aerospace or power plant structures working at relatively high speeds and temperatures, with irregular shapes not being able to undergo typical spin coating. The optical microscopic images showed that the penetration of the sol-gel solution into the composite layers has been quite effective resulting in a crack-free and smooth surface; thus, reducing the porosity, agglomeration, and strengthening the cohesion of the films, which led to a remarkable growth in micro-hardness. The results also expressed the Curie temperature of PZT/PZT samples nearly equal to 300–320 °C, and that of PZT/BiT samples around 370–380 °C, which demonstrates the operational temperature range of such sensors once deposited on jet engine compressor blades as discussed in our previously published paper. Moreover, the sol-gel infiltration showed a great contribution to the improvement of surface roughness, microstructural, dielectric (*C-V*), ferroelectric (*P-E*), and piezoelectric properties of the deposited films. The sensor dimension, number of layers, and porosity exhibited significant impacts on the relative dielectric constant. The dielectric permittivity of PZT/PZT films was observed to be less than that of PZT/BiT ones; the difference may come from a better penetration of thinner BiT sol-gel solution, which consequently helps it to obtain lower porosity. Similar to the dielectric permittivity, the remnant polarization rose by increasing the number of sol-gel infiltrations through the composite layers. The results also depicted a higher remnant polarization in L-PZT/BiT films in comparison with the S-PZT/PZT samples. Similar to the dielectric permittivity and remnant polarization, the piezoelectric strain coefficients had a direct relationship with the thickness, the number of sol-gel infiltration and the film porosity; therefore, the thicker films showed higher piezoelectric coefficients.

## Figures and Tables

**Table 1:** Physical, dielectric and piezoelectric properties of PZT-5A free samples [31].

PZT-5A Zr:Ti = 52:48	Thermal expansion coefficient (CTE) °C	Dielectric constant *ε_r_* @1kHz	Curie temperature (*T_c_*) °C	Density (g/cm^3^)	Di-electric loss *tanδ*	Piezo-electric coefficient *d*_33_ (pm/V)	Piezo electric coefficient *d*_31_ (pm/V)
	3.6×10^–6^	1600	365	7.7	0.02	350	−190
	Longitudinal E/M coupling factor *k*_33_	Transverse E/M coupling factor *k*_31_	Effective E/M coupling factor *k_eff_*	Remnant polarization *P_r_* (μm/cm^2^)	Coercive field *E_c_* (kV/cm)	Saturated polarization *P_sat_* (μm/cm^2^)	
	0.53	0.4	0.5	23	27.7	11.8	

**Table 2:** Micro-hardness values for piezoelectric composite films under 200 g load for 30 s.

Composite Film 28% Porosity PZT/PZT No Infiltration HV(MPa)	PZT/PZT 16% Porosity 30 μm, 5[C+2S] HV(MPa)	PZT/BiT 16% Porosity 50 μm, 5[C+2S] HV(MPa)	PZT 11% Porosity 15 μm (30-layer) HV(MPa)	BiT 9% Porosity 33 μm (30-layer) HV(MPa)
1480	3236	2942	3531	2844

**Table 3:** Mean values of the average roughness (*Ra*) for the piezoelectric thick films (μm).

BiT (~100 nm/Layer)			Composite Film PZT/PZT (No Infiltration)	PZT/PZT 5[C + 2S]	PZT/BiT 5[C + 2S]	PZT (1-Layer)
1-Layer	5-Layer	10-Layer	50 μm	30 μm	50 μm	0.5 μm
0.008	0.015	0.012	7.65	1.757	1.276	0.007

**Table 4:** Characterization results of piezoelectric films over the S and L substrates.

	Pos	*δ* (μm)	Material	Size (mm^2^)	Layers	Porosity (%)	*E_c_* (kV/cm)	*A* (mm^2^)	*ε_r_*	*P_r_* (µC/cm^2^)	*d*_33_ ± 1 (pm/V)
L_1_	A	100 ± 2	PZT/BiT	10 × 10	10	16	20 ± 1	81 ± 1	566 ± 5	23.76 ± 0.01	148
	B	100 ± 2		15 × 15				196 ± 2	410 ± 5	30.01 ± 0.01	135
L_2_	A	60 ± 1	PZT/PZT	10 × 10	10	16	50 ± 1.6	81 ± 1	298 ± 3	16.89 ± 0.01	-
	B	60 ± 1		15 × 15				196 ± 2	154 ± 2	22.37 ± 0.01	
L_3_	A	50 ± 1	PZT/PZT	10 × 10	10	16	60 ± 2.2	81 ± 1	297 ± 3	12.74 ± 0.01	126
	B	50 ± 1		15 × 15				196 ± 2	148 ± 2	10.25 ± 0.01	107
L_4_	A	100 ± 2	PZT/BiT	10 × 10	5	16	20 ± 1	81 ± 1	530 ± 5	21.55 ± 0.01	-
	B	100 ± 2		15 × 15				196 ± 2	393 ± 5	27.17 ± 0.01	
L_5_	A	60 ± 1	PZT/BiT	10 × 10	5	12	20 ± 1.2	81 ± 1	198 ± 2	17.91 ± 0.01	95
	B	60 ± 1							205 ± 2	20.52 ± 0.01	88
L_6_	A	50 ± 1	PZT/BiT	10 × 10	10	12	24 ± 1.5	81 ± 1	193 ± 2	-	-
	B	50 ± 1							191 ± 2		
S_1_	A	22 ± 0.6	BiT	10 × 10	20	9	20.5 ± 2	81 ± 1	77 ± 1	-	-
	C	22 ± 0.6						81 ± 1	82 ± 1		
S_2_	A	50 ± 1	PZT/BiT	15 × 15	5	16	30 ± 1.4	196 ± 2	42 ± 1	5.35 ± 0.01	73
	C	50 ± 1	PZT/PZT	15 × 15			30 ± 1.4	196 ± 2	146 ± 2	6.52 ± 0.01	105
S_3_	A	50 ± 1	PZT/BiT	10 × 10	5	16	30 ± 1.4	81 ± 1	73 ± 1	7.74 ± 0.01	76
	C	50 ± 1						81 ± 1	75 ± 1		
S_4_	A	50 ± 1	PZT/PZT	10 × 10	5	18	30 ± 1.4	81 ± 1	100 ± 1	2.50 ± 0.01	80
	C	50 ± 1						81 ± 1	112 ± 1		
S_5_	C	1000 ± 20	PZT Wafer	*φ* = 10	1000	-	3.5 ± 0.6	71 ± 1	3230 ± 15	-	410
	A	1000 ± 20						71 ± 1	3103 ± 15		410

**Figure A1:**
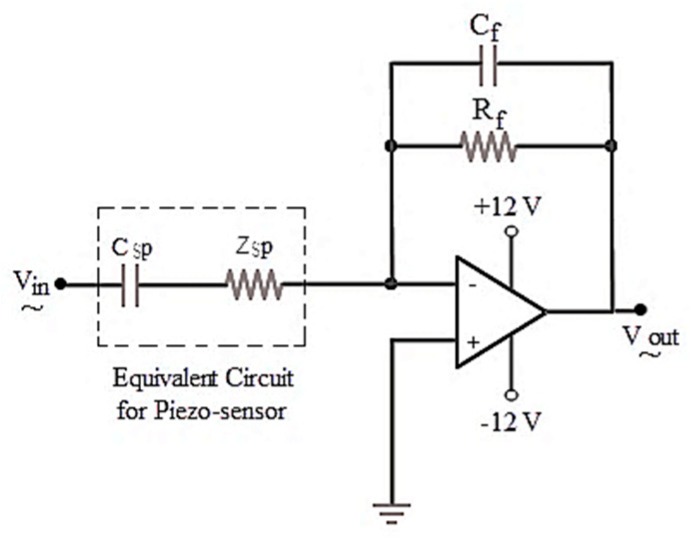
Current to voltage converter circuit for the piezoelectric films undergone the *P-E* hysteresis loop measurement [41].

## Appendix A

% Hysteresis Measurement with a RC Circuit at feedback % C_sp_ = 24.6 nF, R_f_ = 1.83 Mohm, C_f_ = 33 nF; *F* = 50; *T* = 1/f; % Frequency/Period *w* = 2**π***f*; % Input Voltage (*V_in_* applied by the function generator); prompt = {‘V_in_ Amplitude (volts)’, ‘V_out_ Amplitude (volts)’, ‘Phase Difference (ms)’, ‘Feedback Resistance… (Ohm)’, ‘Feedback Capacitance (Farad)’, ‘Sensor Area (mm^2^)’, ‘Film Thickness (mm)’}; defaultanswer = {‘10’, ‘10’,’’, ‘1.83e6’, ‘33e-9’, ‘314.159’, ‘0.2’}; del = inputdlg (prompt,’Input’,1, defaultanswer); V_in__amp = str2num(del{1,:}) % Amplified feed voltage V_out__amp = str2num(del{2,:}) % Output measured voltage Ph_dif = str2num(del{3,:}) % Phase difference R_f_ = str2num(del{4,:}) % Feedback resistance C_f_ = str2num(del{5,:}) % Feedback capacitance A = str2num(del{6,:}) % Piezo-film area thickness = str2num(del{7,:}) % Film thickness time = 0:(*T*/100):*T*; % Time series from digitized data phase1 = -*π*/2; V_in_ = V_in__amp*cos((2* *π* **f* * time)+phase1); % V_in_ vector considering phase difference subplot (3,2,1) plot(time,V_in_,’k’); xlabel(‘TIME (sec)’); % Adding axis labels and plot title ylabel(‘Input Voltage (V)’); title(‘Time Domain Input Voltage’); grid on;
% Output Voltage (*V_out_* measured by the oscilloscope); Ph_rad = Ph_dif*2**π*/(*T**1000); % Convert to Radiant phase_abs = (-Ph_rad+ *π/2*); % Phase difference between V_in_ and V_out_ V_out_ = V_out__amp*exp(-1i*phase_abs)*exp(1i*w*time); subplot (3,2,2) plot(time,V_out_,’b’); xlabel(‘TIME (sec)’); % Add axis labels and plot title ylabel(‘Output Voltage (V)’); title(‘Time Domain Output Voltage’);… (Continued the same as previous) X_c_ = 1/(1i*C_f_*w); % Capacitor impedance Z_f_ = R_f_*X_c_/(R_f_+X_c_); % Feedback Impedance Z_f__amp = sqrt(((real(Z_f_))^2)+(imag(Z_f_))^2); Z_f__phase = atan((imag(Z_f_))/(real(Z_f_))); I_phase = exp(1i*phase_abs)/exp(1i*Z_f__phase) % micro-ampere current = (1e6)*(V_out__amp/Z_f__amp)*(cos((*w**time)+I_phase)); subplot(3,2,3) plot(time, current, ‘r’);
